# The Medical Aspect of the Eastern War

**Published:** 1904-10

**Authors:** 


A Monthly Review of Medicine and Surgery.

EDITOR:

WILLIAM WARREN POTTER, M. D.

All communications, whether of a literary or business nature, books for review and exchanges should be addressed to the editor: 284 FRANKLIN STREET, BUFFALO, N. Y.

VOL. XLIV.—LX. OCTOBER, 1904. No. 3

### The Medical Aspect of the Eastern War.

ASIDE from the general public interest attaching to the great success of Japanese arms in war in the Far Orient over the control of Manchuria, there is a side to the conflict which, while little is said of it in the daily newspapers, has a direct bearing on the morale of the two armies. This is the hygienic and sanitary aspects of the war, and when the methods of both armies are compared, one cannot help feeling a deep sense of pity for the unfortunate Muscovite who is battling beneath the banner of the Bear.

Military experts of all nations have agreed that the Japanese army is far and away the better fighting machine today, due perhaps to the preparedness with which it entered the conflict. Without going into the detail of official inefficiency and bureaucratic corruption, which has been credited to Russia, and which has no proper place in these pages, a brief résumé of the methods of the opposing forces in dealing with hygienic and sanitary affairs, field treatment of wounds, transportation facilities and hospital equipment, will, in a measure, explain to the medical man some of the discouraging defeats which Kouropatkin has suffered. A sick army is worse than no army at all; an army poorly fed, illy-clothed and cognisant of the horrors of medical neglect is really worse than a sick army.

The Japanese surgeons prepared for war in times of peace. They took part in the annual maneuvers; not as sightseers, but as workers. Hospital tents were erected and relief stations established. Supposedly wounded men were received and prepared for operation for every wound probable or possible in actual warfare. The instruments were actually sterilised, the solutions were mixed with full strength drugs and chemicals and dressings were made ready, and the forms of operation were actually gone through with, dressings applied, the wounded man being carried to his cot and watched as if he were really under an anesthetic. Sick diet was prepared and even administered; transportation of sick and wounded was carried out and all patients were properly recorded and never lost sight of at any time. The supposedly dead men were removed without delay from hospitals and placed in dead house tents where preparations were made for incineration of the bodies. In fact, the whole system of efficient, rapid and intelligent care of sick and wounded was gone over, and each man in the medical department from the chief surgeons to the humblest orderly was made ready.

The apparent waste of material in preparation called forth from an English surgeon the remark that such waste was useless in mere rehearsal. This was replied to by the Japanese surgeon-general in an epigram, terse and pregnant of meaning:

"Nothing is wasted which is conducive to the health and comfort of a Japanese soldier."

Now, in real warfare the Japanese medical department is an efficient, smoothly-working machine, and the soldier is getting the full benefit of the "waste" in time of peace. Further, the medical department of the army has left behind it a path free from epidemic. The dead are incinerated on the field of battle and their ashes boxed and sent to Japan for burial. The burning is done by Chinese coolies under the direction of a Japanese medical officer, and oil is the fuel used. That is a pen picture of the methods in the field, painted by a race that has been looked upon for years as "heathen."

See now, how the enlightened white man of Russia, the great and ever-ready, deals with similar problems. They grew careless in their might. The wounded and sick of the Russian forces were shipped back from the front, first to Liao-yang; then to Mukden, and now are being deserted on the field for lack of transportation facilities. There may be some film of excuse for this desertion because the Russian army is harassed by a tenaceous and persistent foe and in the recent days the Bear has had to travel fast, and sick and wounded are impedimenta which hamper rapid movements. Yet before this unseemly haste was a necessity the Russian wounded and sick were given scant care. They received such aid as may be on the field or at temporary dressing stations at which little provision had been made for more than a mere skirmish in many cases. The hospital bases were not connected with the firing line or with headquarters by wire and hence had no information of the shipment of large numbers of sick and wounded, who were dumped in on them unawares and unprepared for.

Into Mukden came the sick and wounded later, by authority of newspaper correspondents, packed in dirty box cars, lying on the bare floors without even a blanket; the majority of the wounded were as they had been taken from the field; their clothing in rags and dirt encrusted; their wounds open and exposed, or mayhap tied up with a kerchief or a rag of clothing; the wounded huddled in with the sick, the greater number of whom were suffering with that dread army disease—dysentery. This condition was bad enough, the packing of sick and wounded indiscriminately into box cars and dirty ones, at that; but there were absolutely no sanitary provisions made; there were no steps to the cars—the sick and wounded were practically prisoners. Even at way stations where the trains were held for the passage of troop transport trains hurrying to and from the front, the sick were not allowed the luxury of the use of the foul little closets, but were kept in the cars. It does not need a very active imagination to picture the condition of these poor wretches of the Czar's soldiers on their arrival at their destination—the base hospital—only to find that there had not been provision made for their reception, and that they were compelled to remain an indefinite time in the cars until hasty preparations could be made for their reception as patients.

The latest phase of incompetency on the part of the Russians adds to the horror of a corrupt and illy-administered campaign. The medical department is not to blame. It is doing what it can with what it has. The vast sums of money which have been appropriated for hospital use and medical supplies have been stolen by the bureaucrats, the open charge having been made in newspapers in different parts of the world that one of the grand dukes of the imperial family had diverted to his own use the money which came into his possession for the purchase of medical equipment.

It is no wonder the Japanese soldier can fight for twelve or fifteen days at a stretch, cover wide ranges of territory and go into battle again apparently as fresh as at the beginning, while the Russian soldier is fagged and sick and discouraged by defeat on defeat. The Japs constitute a healthy army; the Russians, a sick army.

We have not written in a spirit of prejudice, but have aimed simply to give a general summary of the facts as delineated in the medical and lay press.

